# Mechanism of jianxin granules in the treatment of heart failure based on proteomics and metabolomics

**DOI:** 10.1186/s13020-024-01009-6

**Published:** 2024-11-28

**Authors:** Chen Yongzhong, Chen Hui, Zhang Luting, Guo Wei, Huang Yiqing, Guo Yiru, Su Linqiu, Xu Rong, Li Xi, Ouyang Qiufang

**Affiliations:** 1 Gerontology Department, The Second Affiliated Hospital of Fujian University of Traditional Chinese Medicine, Fuzhou, 350003 Fujian China; 2 The First Affiliated Hospital of Guangzhou University of Chinese Medicine, Guangzhou University of Chinese Medicine, Guangzhou, 510006 Guangdong China; 3 Ultrasound Department, The Second Affiliated Hospital of Fujian University of Traditional Chinese Medicine, Fuzhou, 350003 Fujian China

**Keywords:** Heart failure, JX granules, Proteomics, Metabolomics, Pyruvate metabolism, PI3K/Akt signaling pathway

## Abstract

**Background:**

Heart failure (HF) is associated with high mortality and rehospitalization rates, highlighting the need for novel therapeutic approaches. Jianxin (JX) granules, a Traditional Chinese Medicine formulation, have been patented for the treatment of HF. However, the specific therapeutic effects and underlying mechanisms of JX granules have not been fully elucidated. This study aimed at investigating the effects and mechanism of JX granules in the treatment of HF based on proteomics and metabolomic profiling.

**Methods:**

HF model was established in rats by ligation of left coronary artery. The successfully modeled rats were randomly divided into three groups: the model group, the JX granules group, and Sacubitril/Valsartan (S/V) group. Four weeks after treatment, left ventricular (LV) function was evaluated via echocardiography. LV fibrosis and apoptosis were examined through histological analyses, while mitochondrial morphology was assessed using transmission electron microscopy. Quantitative assessment of oxidative stress was also conducted. Proteomics was used to identify the differentially expressed proteins and potential pathways. Metabolomics was utilized to elucidate the variations in metabolism. Then western blotting and in vitro analyses were performed.

**Results:**

A rat model of HF was established, evidenced by a decrease in left ventricular ejection fraction (LVEF), stroke volume (SV), and left ventricular fractional shortening (LVFS), alongside diminished adenosine triphosphate (ATP) content, elevated oxidative stress, augmented apoptosis, and disrupted pyruvate metabolism. Treatment with JX granules ameliorated these effects, improving systolic function, reducing ventricular chamber size, and increasing LVEF, SV, and LVFS, as assessed by echocardiography. Additionally, JX granules attenuated cardiac fibrosis and improved mitochondrial structure, as evidenced by less vacuolation and clearer mitochondrial cristae, when compared to the model group. The treatment also regulated apoptosis-related protein expression, partially reversing the increase in cleaved Caspase-9, cleaved Caspase-3, and Bax and the suppression of Bcl-2 observed in the heart failure rats. All of these effects were similar to S/V. Proteomic and metabolomic analyses identified key differential genes, such as triosephosphate isomerase 1 (TPI1), lactate dehydrogenase B (LDHB), pyruvate kinase M (PKM), protein kinase B (Akt), Pyruvate Dehydrogenase Beta (PDHB) and lactate dehydrogenase A (LDHA), as well as vital pathways including carbon metabolism, the PI3K-Akt signaling pathway, pyruvate metabolism, and HIF-1α signaling pathway. Moreover, JX granules mitigated oxidative stress, inhibited apoptosis, and activated Akt in H9c2 cells exposed to angiotensin II, which could be reversed by the PI3K inhibitor LY294002.

**Conclusion:**

JX granules improve HF in parallel to the efficacy of S/V, at least in part, through enhancing pyruvate metabolism, inhibiting oxidative stress and activating PI3K/Akt pathway.

**Supplementary Information:**

The online version contains supplementary material available at 10.1186/s13020-024-01009-6.

## Introduction

Heart failure (HF), is a clinical syndrome resulting from persistent cardiac pump dysfunction. It is associated with high rate of prevalence, mortality and rehospitalization [1]. Myocardial infarction has been the most common cause of heart failure [2]. Despite significant advances in current treatments, the 5-year mortality rate in HF patients remains unacceptably high. Therefore, it is imperative to explore the novel therapeutic approaches for HF.

The pathogenesis of HF is complicated and multifactorial. Emerging evidence suggests that oxidative stress and phosphatidylinositide 3-kinase (PI3K) play a pivotal role during the transition from ischemia to HF [3]. HF is associated with increased oxidative stress, leading to mitochondrial dysfunction and a reduction in ATP production [4]. Additionally, key signaling pathways, such as PI3K and its downstream protein kinase B (Akt) are involved in the disease mechanism [5]. Consequently, targeting the regulation of oxidative stress and PI3K/Akt pathway offers promising therapeutic approaches for HF.

Jianxin (JX) granules, a traditional Chinese medicine formulation used in hospitals, have been patented to treat HF [6]. JX granules contain Huang Qi (*astragalus*), Hong Shen (*red ginseng*), Pu Huang (*pollen typhae*), Dan Shen (*salvia miltiorrhiza*), Zhu Ling (*polyporus*), Bai Zhu (*atractylodes macrocephala*), Gui Zhi (*cassia twig*), Ting Li Zi (*semen lepidii*). Modern pharmacological studies have revealed that JX granules can significantly improve the symptoms of heart failure and reverse ventricular remodeling in patients with HF [7]. Another study also indicated that JX granules can improve myocardial energy metabolism in HF rats [8]. However, the specific therapeutic effects and underlying mechanisms have not been fully elucidated yet, due to the complexity of the components of JX granules and the lack of high-performance analytical methodologies.

In recent years, omics technologies, particularly proteomics and metabolomics, have experienced significant advancements. These methodologies offer a powerful tool for identifying disease-specific biomarker and uncovering systemic metabolic targets. Consequently, they provide a method for investigating the mechanisms of traditional Chinese medicine [9].

This study investigates the effects of JX granules on HF and elucidates potential underlying mechanisms through proteomic and metabolomic analyses. In particular, we focus on oxidative stress and PI3K/Akt-mediated apoptosis pathways to uncover their roles in the therapeutic impact of JX granules.

## Material and methods

### Animal model establishment and experimental protocol

All procedures and protocols were approved by the Institutional Animal Care and Use Committee of Fujian University of Traditional Chinese Medicine. Forty healthy male Sprague–Dawley (SD) rats (190–210 g) were obtained from Beijing Huafukang Biotechnology Co., Ltd (China). The rats were housed in standard laboratory condition, which included a 12-h light/dark cycle, a controlled temperature of 25 ℃, and a relative humidity of 60%. They were offered with ad libitum access to standard food and distilled water. After a 7-day adaptation period, the rats underwent experimental modeling.

The HF model was established according to the previous literature [10]. All the rats were anesthetized using isoflurane (5 ml/min × 5% initially, followed by maintenance at 3 ml/min × 3%; Shenzhen Rayward Life Technology Co., Ltd). The ligation of the left anterior descending (LAD) artery resulted in immediate myocardial infarction onset. Left ventricular function was assessed by echocardiography one-week post-infarction. During this period, the animals did not receive any pharmacological treatment and their diet of standard lab chow and distilled water was maintained as before. Rats with a left ventricular ejection fraction (LVEF) below 50% were included in the subsequent study. They were randomly assigned to the model group, JX granules group, and Sacubitril/Valsartan (S/V) group. The sham group functioned as the negative control. S/V was utilized as the positive control due to its supremacy over other traditional HF treatments and its multifaceted mechanism of action. Its efficacy is well-documented in clinical literature [11]. Moreover, it modulates various pathological factors related to heart failure, such as fibrosis, oxidative stress, apoptosis, and PI3K/Akt pathway, etc. [12].

The control group and the model group were administered 2 ml normal saline by gavage. The herbal components of JX granules were provided by Second People’s Hospital Affiliated to Fujian University of TCM. According to the pharmacological experimental methodology (4th Edition) and the formulas for its equivalent to clinical doses (30 mg/d), the dose for rats is calculated as 6.3X mg/kg, where 6.3 represents the conversion factor; X indicates effective dose for humans; 60 kg is the average human weight [13]. In the study, the rats were administered JX granules at the dosage of 6.3 × 30 mg/60 kg, that is 3.2 g/kg. Rats in the Sacubitril/Valsartan (S/V) group were treated with S/V at a daily dosage of 68 mg/kg [14].

Following a 4-week intervention, LV function was assessed using echocardiography. Then the rats were euthanized via cervical dislocation following anesthesia induction with 2.5% isoflurane. LV tissue samples were collected for subsequent experiments.

### Echocardiographic evaluation of left ventricular function

All rats were subjected to echocardiographic evaluation as previously described [15]. In brief, under Isoflurane anesthesia (2%) administered via a face mask, LV function was assessed using an ultrasound machine (PHILIP EPIQ 7) equipped with an 18-MHz transducer. Various ultrasound parameters, including interventricular septal thickness rate (IVS), posterior wall thickness rate (LVPW), end-diastolic and systolic left ventricular diameter (LVIDd and LVIDs), as well as LV ejection fraction (LVEF), fractional shortening (LVFS), and stroke volume (SV), were determined through M-mode echocardiography. These measurements were averaged over three successive cardiac cycles.

### Histological examination staining

Histological staining was conducted to examine morphological alterations, myocardial fibrosis, ultrastructural changes, and myocardial apoptosis. Briefly, left ventricular samples were isolated and weighed. Myocardial sections from the mid-papillary muscle level underwent hematoxylin–eosin (HE) staining, Masson staining, electron microscopy, and terminal deoxynucleotidyl transferase-mediated dUTP nick-end labeling (TUNEL) assay. Regarding the experiments in vitro, H9c2 cells were fixated with 4% paraformaldehyde, followed by similar procedures as LV sections. The apoptosis index, denoting the number of TUNEL-positive cardiomyocyte nuclei as a percentage of the total cardiomyocyte nuclei, was calculated [16].

### Colorimetric measurement of enzyme activities

Adenosine triphosphate (ATP), total antioxidant capacity (T-AOC), malondialdehyde (MDA) activity, and reactive oxygen species (ROS) were assessed via colorimetric assays as per the manufacturer’s protocols (Nanjing Jiancheng, China). Homogenized samples from the border zone of the fresh LV myocardial infarction and H9c2 cell lysate were utilized. Absorbance readings were obtained at 636 nm for ATP, 405 nm for T-AOC, and 532 nm for MDA measurements. Reactive oxygen species (ROS) levels were determined by measuring absorbance at an excitation wavelength of 488 nm and an emission wavelength of 525 nm. Additionally, LV frozen sections or cells were incubated with DCFH-DA fluorescence. The fluorescence intensity denoted the superoxide levels of tissue in situ.

### Western blot analysis

LV samples or H9c2 cells were lysed. The protein concentration was determined. The quantitative protein samples (20 μg) were subjected to SDS-PAGE and then moved to a PVDF membrane (Millipore, Bedford, MA, USA). The membrane was incubated with the corresponding primary antibodies including Akt and p-Akt, PI3K and p-PI3K, cleaved-caspase-9, caspase-9, cleaved-caspase-3, caspase-3, LDHA, PKM2, HIF-1α, Bcl-2, bax, PDHB, and GAPDH (Abcam, USA). Then the membrane was incubated with a secondary antibody. Subsequently, proteins were visualized with Phototope-HRP detection kit (Cell Signaling Technology). Finally, western blot signals were scanned and the optical density of samples were analyzed.

### Proteomics

For each sample, 2 µg of total peptides were separated and analyzed with a nano-UPLC (EASY-nLC1200) coupled to a Q Exactive HFX Orbitrap instrument (Thermo Fisher Scientific) with a nano-electrospray ion source. Data dependent acquisition (DDA) was performed in profile and positive mode with Orbitrap analyzer. Vendor’s raw MS files were processed using Proteome Discoverer (PD) software (Version 2.4.0.305) and the built-in Sequest HT search engine. MS spectra lists were searched against their species-level UniProt FASTA databases (uniprot-Rattus norvegicus-10116-2021-8.fasta), with carbamidomethyl [C], TMT Pro (K) and TMT Pro (N-term) as fixed modifications, whilst Oxidation (M) and Acetyl (Protein N-term) as variable modifications. Peptide identification utilized an initial precursor mass deviation of up to 10 ppm and a fragment mass deviation of 0.02 Da for accuracy. Unique peptide and Razor peptide were utilized for the protein quantification, while total peptide amount enabled data normalization.

Differentially expressed proteins were identified based on a 1.5-fold change cutoff, combined with a statistical significance criterion (p-value < 0.05) from a t-test. This ensured that only proteins showing both biological and statistical relevance were considered as differentially expressed. Inner-quartile data from control experiments were analyzed using ln-ln plots. Multivariate analyses, including 2D Hierarchical Cluster Analysis (HCA) Heatmaps and Principal Component Analysis (PCA) plots, were performed using Qlucore Omics Explorer (Qlucore, Lund Sweden). Gene ontology assignments and the pathway analysis were executed with MetaCore (GeneGO Inc., St. Joseph, MI, USA).

### Metabolomics

LV samples were initially collected and equilibrated at 4 °C. Subsequently, 1 ml of a mixture containing acetonitrile, methanol, and ddH_2_O (in a 2:2:1 ratio, v/v/v) was added to the plasma samples. Afterward, the samples were centrifuged at 4 ℃ for 10 min at 12,000 rpm. The dissolved samples were then vortex-mixed and centrifuged once more. Finally, the prepared samples were filtered through a 0.22 µm membrane for LC–MS analysis.

The analysis of these LV samples was conducted using the Thermo Scientific Vanquish UHPLC-Q Exactive system with a Hyperil Gold C18 column. The stability of ESI-MSn experiments was assessed using a Thermo Q Precision mass spectrometer with a spray voltage of 3.2 kV set for both positive and negative ionization modes. Additionally, the sheath gas flow rate ranged from 10 to 40 arb, and the capillary temperature was maintained at 320 °C. An HCD scan was employed to enable data-dependent acquisition (DDA) MS/MS experiments. Redundant information within the MS/MS spectra was further eliminated through dynamic exclusion.

Combined metabolomic and proteomics analysis was performed. |Fold change|> 1.5 was used as the threshold to screen for differentially expressed proteins and metabolites, and then they were imported the filtered data into the Venny tool for visualization of the intersections between the datasets. The common pathway analyses specific to the Rattus norvegicus species were conducted using Kyoto Encyclopedia of Genes and Genomes (KEGG) pathway database (www.kegg.jp/kegg/pathway.html).

### Cell culture and treatment, as well as the analysis of ROS, apoptosis, akt activity, and ATP production

H9c2 cells were cultured with Ang II at a dose of 100 nmol/l for 24 h to induce apoptosis [17]. JX granules drugged serum (500 μg/ml) or PI3K inhibitor LY294002 (10 μM) was administered to H9c2 cells 1 h before exposure to Ang II, and the cells were exposed to these treatments for 24 h.

ROS production was tested using dichlorofluorescein diacetate (DCFH-DA) staining. Cells treated with Ang II for 48 h were incubated with DCFH-DA. Apoptosis was assessed by TUNEL staining. The protein expressions of Akt and p-Akt were determined by western blotting. And the concentrations of ATP in those cells were determined.

### Data analysis

The heatmap analysis and KEGG enrichment analysis were carried out using Majorbio Cloud Platform. Pathway analysis was performed using Metabo Analyst toolset. The data were presented as mean ± SD and were statistically analyzed with GraphPad Prism 6.0 (GraphPad Software Inc., CA, USA). Statistical comparison was made through one-way analysis of variance (ANOVA), followed by post hoc Bonferroni tests. A two-tailed p-value below 0.05 was considered statistically significant.

## Results

### JX granules improved LV function and inhibited cardiac fibrosis in rats with HF

During this study, there were 4 deaths in the model group, 2 deaths in the JX granules group, and 1 death in the S/V group. Notably, there were no deaths in the control group. The weight of rats and left ventricular mass showed no significant difference among the groups (Table 1). Compared to the control group, the rats in the model group showed reduced myocardial systolic amplitude (thickening of IVS and LVPW), enlarged ventricular chamber (LVIDs and LVIDd) and decreased systolic function (lower LVEF, SV, LVFS). JX granules treatment enhanced systolic motion amplitude, reduced ventricular chambers, as well as increased LVEF, SV and LVFS. There was no statistically significant difference between JX granules group and S/V group.

**Table 1:** Left ventricle mass and echocardiography parameters among the groups

	Control	Model	JX granules	S/V
n	10	6	8	9
Final body weight (g)	538.58 ± 32.52	456.18 ± 64.76	507.65 ± 72.40	487.24 ± 58.51
Left ventricle mass (g)	1.25 ± 0.09	1.65 ± 0.31	1.45 ± 0.25	1.41 ± 0.18
IVSd (mm)	2.06 ± 0.15	1.35 ± 0.24^*^	1.46 ± 0.31^#^	1.58 ± 0.29^#^
IVS Thickening (%)	47.4 ± 3.0	20.1 ± 4.18^*^	26.04 ± 3.19^#^	37.2 ± 4.75^#^
LVPWd (mm)	2.01 ± 0.25	1.95 ± 0.30	1.89 ± 0.36	1.93 ± 0.28
LVPW thickening (%)	49.5 ± 4.26	13.7 ± 3.07^*^	34.6 ± 5.38^#^	40.06 ± 3.72^#^
LVIDd (mm)	5.95 ± 0.73	8.01 ± 1.06^*^	6.83 ± 0.76^#^	6.71 ± 0.90^#^
LVIDs (mm)	3.45 ± 0.38	7.19 ± 0.92^*^	5.16 ± 0.46^#^	4.85 ± 0.58^#^
LVEF (%)	78.15 ± 5.67	31.28 ± 6.30^*^	56.74 ± 9.58^#^	62.34 ± 10.34^#^
SV (ml)	0.41 ± 0.05	0.22 ± 0.04^*^	0.31 ± 0.07^#^	0.36 ± 0.05^#^
LVFS (%)	41.57 ± 3.04	15.29 ± 3.19^*^	26.74 ± 4.58^#^	30.52 ± 3.71^#^

Data were expressed mean ± standard deviation

*IVSd* interventricular septum at diastole, *IVS* interventricular septum, *LVPWd* left ventricular posterior wall at diastole, *LVIDd* left ventricular diastolic internal dimension, *LVIDs* left ventricular systolic internal dimension, *LVEF* left ventricular ejection fraction, *SV* stroke volume, *LVFS* left ventricular fractional shortening. *p < 0.05 vs the control group, ^#^*p* < 0.05 *vs* the model group

**Fig. 1:**
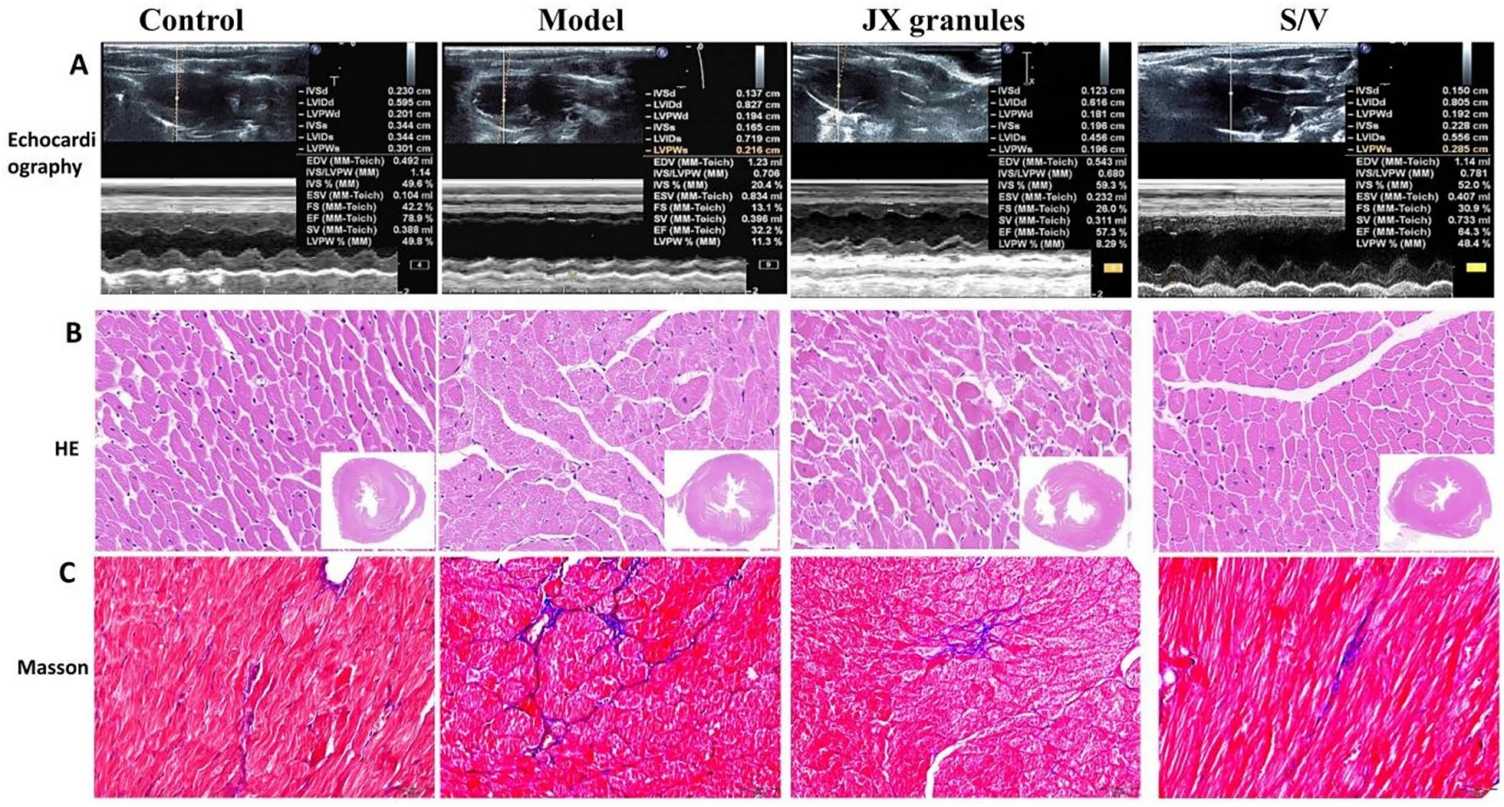
Effects of JX granules on left ventricular histopathology in rats with HF. **A** Representative M-mode echocardiographic images. **B** Representative images of HE staining. **C** Representative images of Masson trichrome staining

As compared with the control group, the rats in the model group displayed enhanced septal echogenicity and reduced IVS thickening. JX granules or S/V treatment enhanced IVS motion amplitude (Fig. 1A). The rats in the control group had regular myocardial cell morphology, arranged tightly and orderly, and no edema was observed (Fig. 1B). And the model rats showed disordered myocardial cell arrangement, cardiomyocyte hypertrophy or necrosis, interstitial edema, and collagen fiber hyperplasia. However, the rats in the JX granules and S/V groups showed more orderly myocardial cell arrangement by comparison with the model group. Additionally, LV myocardium in the model group exhibited increased collagen fiber (blue) as compared with the control group (Fig. 1C), which indicating cardiac remodeling. And fibrosis was significantly attenuated by JX granules or S/V treatment.

### JX granules ameliorate mitochondrial injury and improve oxidative stress in HF rats

**Fig. 2:**
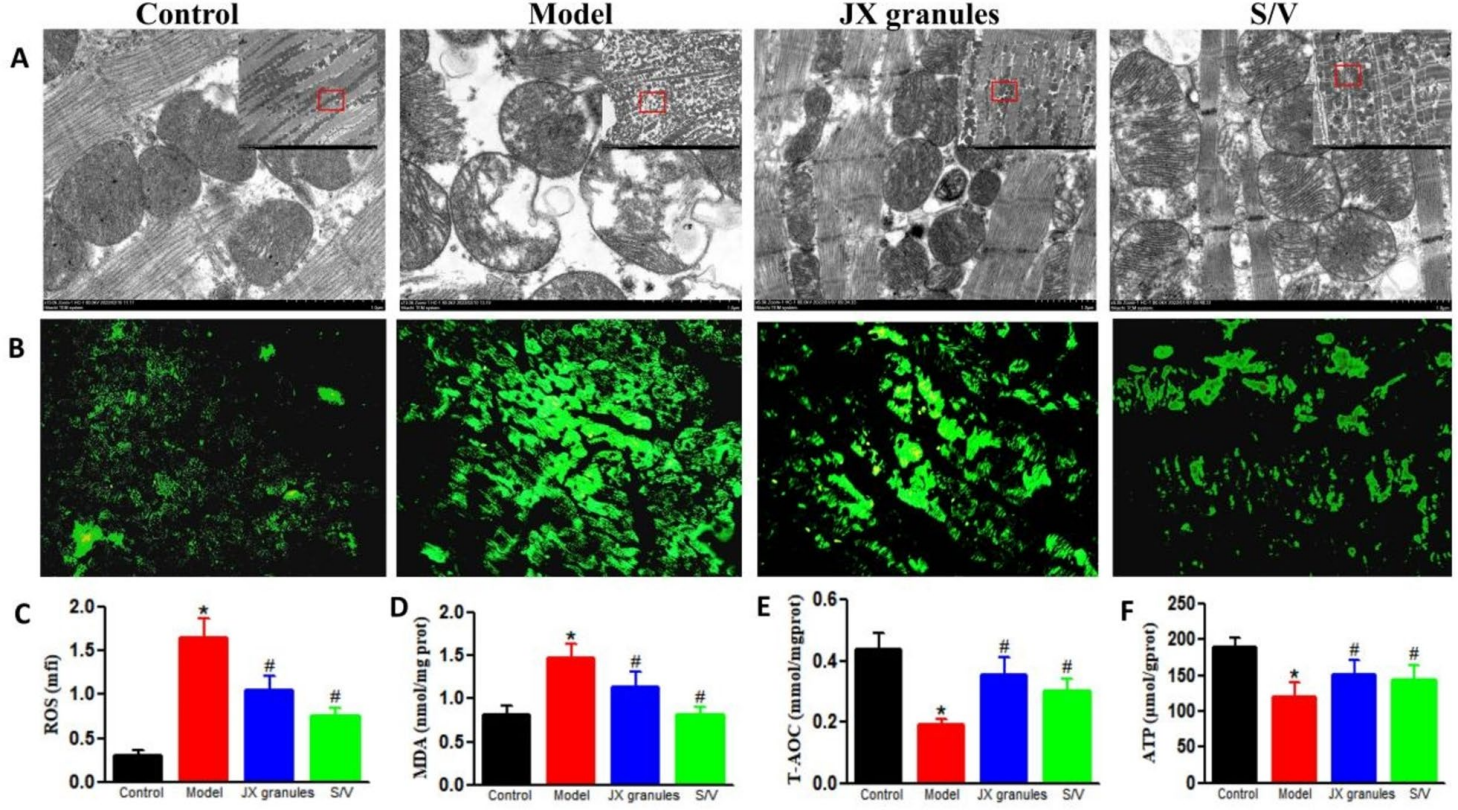
Effects of JX granules on mitochondrial structure and oxidative stress in HF rats. **A** Representative images of mitochondrial structure, depicted by transmission electron microscopy. **B** Reactive oxygen species (ROS) levels in LV myocardium detected by DCFH-DA staining. **C** Relative DCFH-DA fluorescence intensity for ROS. **D**–**F** The levels of T-AOC, SOD, and ATP in myocardium determined by specific assay kits. Data were expressed as mean ± SD; ∗*p* < 0.05 versus control group; ^#^*p* < 0.05 versus model group

To elucidate the effect of JX granules on mitochondrial function, the mitochondrial morphology, oxidative stress, and ATP levels in myocardium were analyzed (Fig. 2).

The mitochondrial cristae in the control group were clear, almost no mitochondrial vacuolization occurred. In the model group, mitochondria showed swelling, disorder of sparse cristae, fracture, and vacuoles. And the mitochondria in JX granules and S/V groups were slightly swollen with less vacuolation and more visible mitochondrial cristae.

The concentration of ROS and MDA increased in HF rats as evidenced by increased fluorescence intensity. Meanwhile, the antioxidant capability (T-AOC) significantly decreased. JX granules and S/V partially reversed the changes of oxidative stress.

The levels of ATP decreased in LV myocardium of model rats as compared to control rats (120.7 ± 20.1 vs 190.5 ± 12.1, p < 0.05). In contrast, they were increased by 0.26-fold following JX granules or by 0.19-fold after S/V treatment.

### JX granules inhibited apoptosis in HF rats

**Fig. 3:**
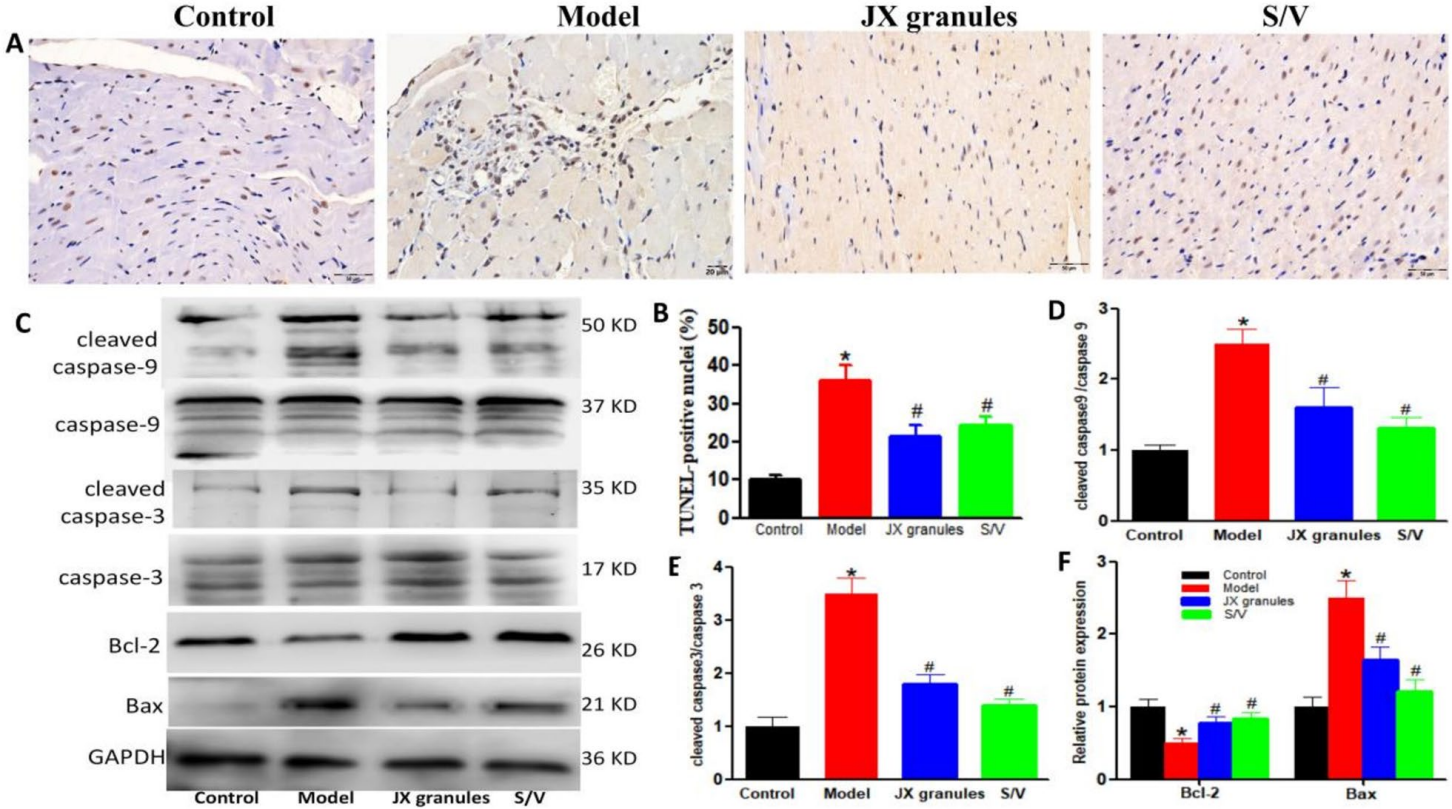
Effects of JX granules on apoptosis in HF rats. **A** TUNEL (TdT-mediated dUTP Nick-End Labeling) assay for apoptosis detection. Brown nuclei indicated cell apoptosis. **B** The apoptotic index in LV myocardium. **C**–**F** The protein expressions of caspase-9, cleaved-caspase-9, caspase-3, cleaved-caspase-3, Bcl-2, and Bax were analyzed by western blotting. The protein densitometry was normalized with caspase-3, caspase-9, or GAPDH. And they were quantified of three experiments. ∗*p* < 0.05 versus control group, ^#^ *p* < 0.05 versus model group

Myocardial apoptosis was determined by TUNEL (Fig. 3A). In control rats, myocardial nuclei were stained blue (normal nuclei). And scarcely brownish yellow nuclei (apoptotic nuclei) were observed. The percentage of apoptotic cells increased significantly in the model group (10.25 ± 2.16%) as compared to the control group (35.43 ± 3.40%), which was reversed by JX granules (24.78 ± 3.11%) or S/V treatment (28.12 ± 2.85%). Western blot assay was performed to examine the apoptosis-related molecules. The elevated expression of cleaved Caspase-9/Caspase-9, cleaved Caspase-3/Caspase-3, and Bax in model rats, all of which were partly reversed upon treatment with JX granules or S/V. Conversely, the expression of Bcl-2 was suppressed in HF rats but exhibited an increase following treatment with JX granules or S/V.

### Effects of JX granules on differential expressed proteins in HF rats

**Fig. 4:**
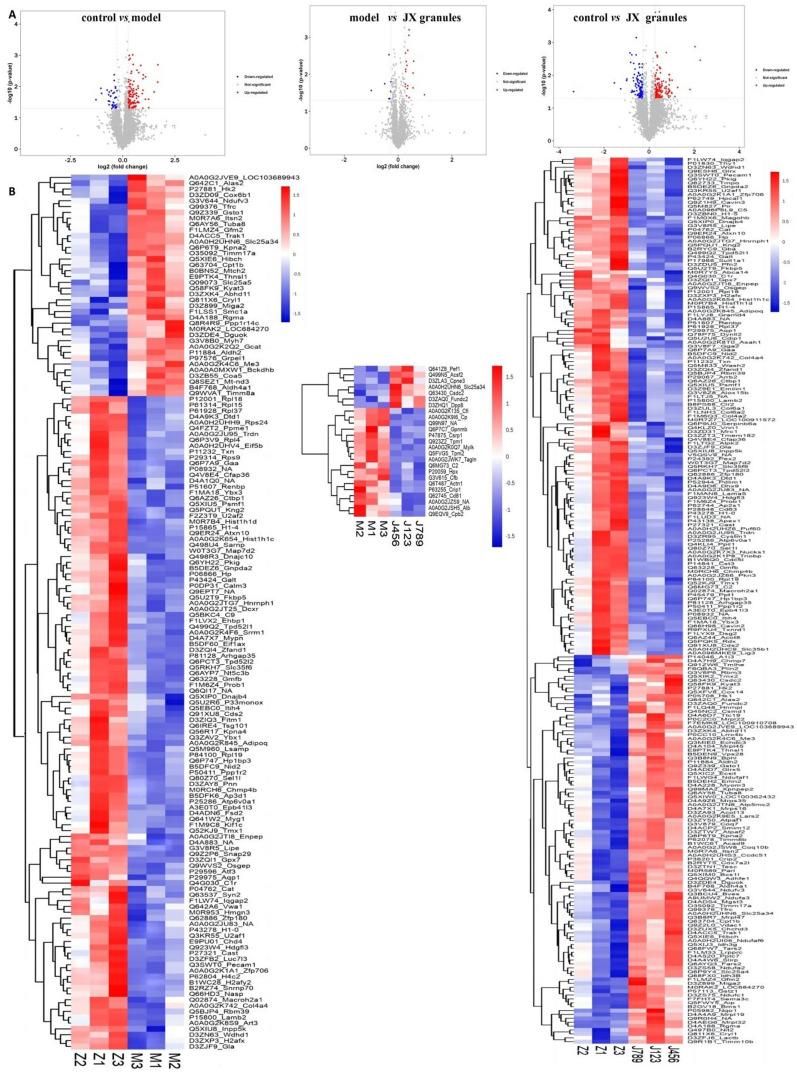
Effects of JX granules on differentially expressed proteins in HF rats. The volcano plots (**A**) and Heatmaps (**B**) of differentially expressed proteins.Their expression is calculated by log2 method and displayed in the volcano plots and heatmaps in different colors, where red represents significantly up-regulated proteins, blue represents significantly down-regulated proteins, and gray part represents no quantitative information of proteins. *DEPs* differentially expressed proteins, *M* model group, *J* JX granules, *Z* control group

The differential expressed proteins (DEPs) were obtained with Tandem Mass Tags approach. As compared to the control group, 150 DEPs were identified in model rats, comprising 112 up-regulated and 38 down-regulated proteins. Notably, in the JX granules group, 25 DEPs were identified compared to the model rats, encompassing 18 up-regulated and 7 down-regulated proteins. In comparison with the control group, 228 DEPs in the JX granules group were obtained, which comprised of 128 up-regulated and 100 down-regulated proteins. All DEPs data were illustrated with volcano plots and heatmaps (Fig. 4).

**Fig. 5:**
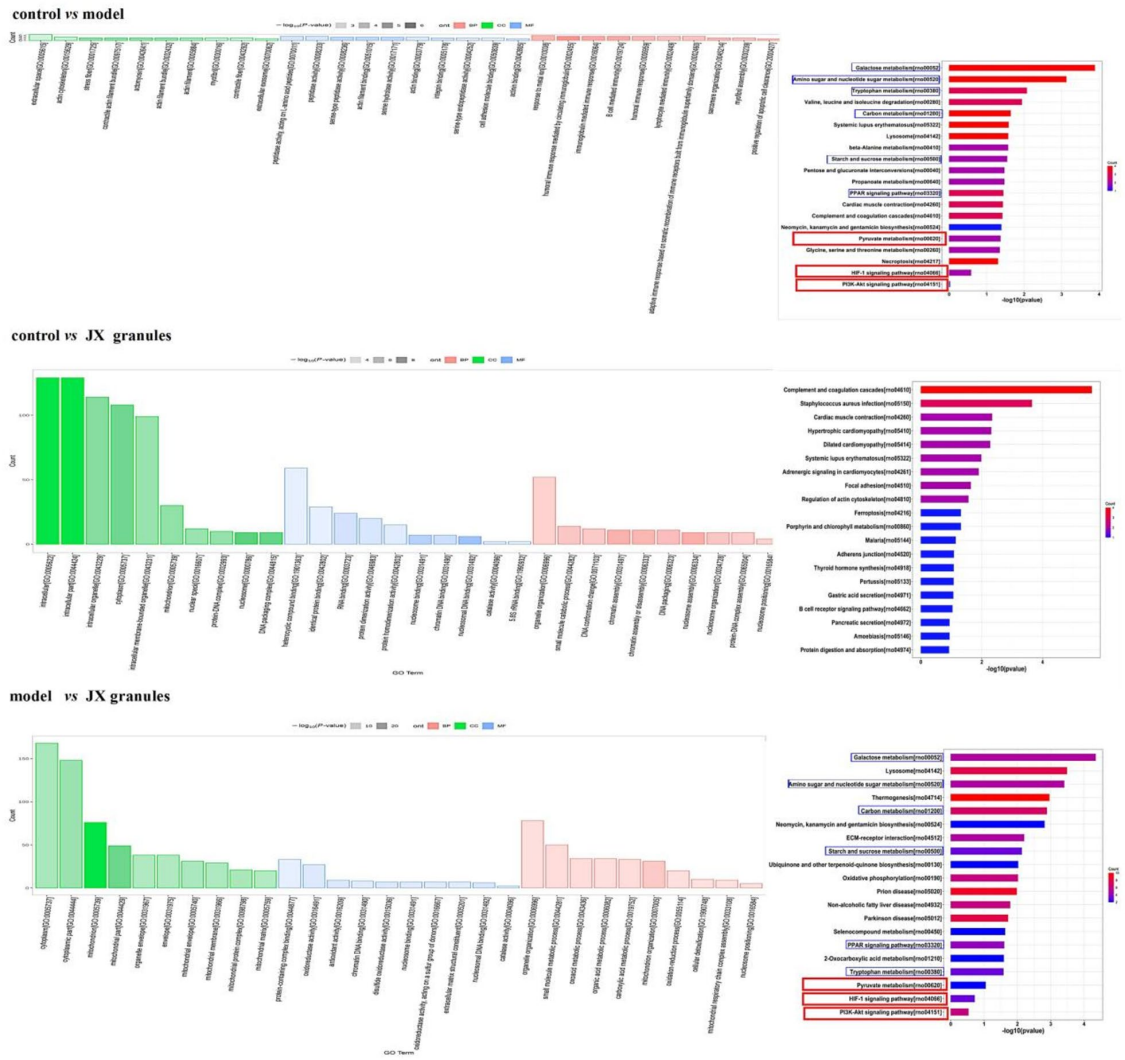
Effects of JX granules on signal pathway. The GO functional and KEGG enrichment of differentially expressed proteins. *GO* Gene Ontology, *KEGG* Kyoto Encyclopedia of Genes and Genomes, *BP* biological processes, *MF* molecular function, *CC* cell composition

GO enrichment analysis of biological process (BP), cellular component (CC), and molecular function (MF) components, as well as the KEGG enrichment pathways were explored (Fig. 5). As compared with the control group, BP in the model group was mainly enriched in humoral immune response, positive regulation of apoptotic cell clearance, and the response to metal ions; Meanwhile, CC was mainly related to contractile and stress fibers, actin filaments, and extracellular space; MF was mainly enriched in the serine-type peptidase activity, actin binding, and peptidase activity. Furthermore, DEPs indicated by KEGG analysis were primarily linked to pathways such as the complement and coagulation cascades, regulation of the actin cytoskeleton, and adrenergic signaling. Comparative KEGG pathway analysis between the control and model groups indicated that DEPs were predominantly enriched in galactose metabolism, amino sugar and nucleotide sugar metabolism, and tryptophan metabolism. Similarly, the analysis comparing the model group with JX granules group highlighted significant enrichment in pathways such as galactose metabolism, lysosome, and amino sugar and nucleotide sugar metabolism. In contrast, the comparison between the control group and the JX granules group revealed enrichment in the complement and coagulation cascades, cardiac muscle contraction, and adrenergic signaling in cardiomyocytes. The KEGG analysis identified several pathways commonly affected in both the control/model and model/JX granules groups, including galactose metabolism, amino sugar and nucleotide sugar metabolism, tryptophan metabolism, carbon metabolism, starch and sucrose metabolism, PPAR signaling pathway, pyruvate metabolism, HIF-1α signaling, and the PI3K-Akt pathway.

### Effects of JX granules on differential metabolites in HF rats

**Fig. 6:**
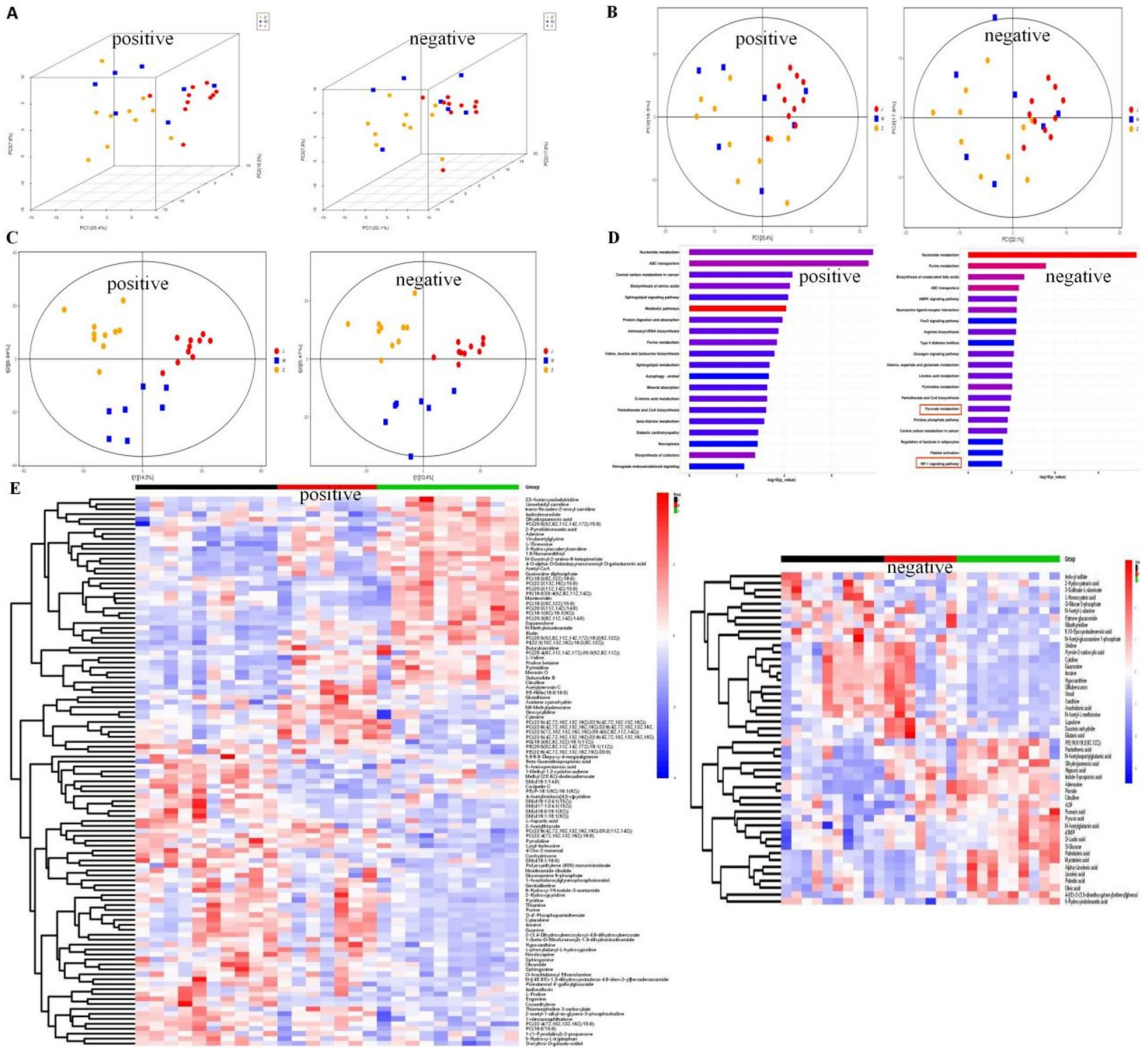
Effects of JX granules on differential metabolites in HF rats. Metabolomics analysis identified possible for JX granules. **A** PCA-3D, **B** PCA and **C** PLS-DA analysis in the positive and negative modes. The different colors represent the different groups (red for the control, blue for model, purple for JX granules. **D** KEGG analyze the metabolic the metabolic pathway of the potential biomarkers in the negative and positive modes. **E** Heat map analysis of 110 and 49 differential metabolites in the positive and negative modes

To further investigate the effects of JX granules on metabolic processes in HF rats, the differential metabolites in myocardial tissue were examined. Multifaceted approaches including 3D-PCA analysis (Fig. 6A), PCA analysis (Fig. 6B), PLS-DA (Fig. 6C) were employed to discern the distinct clustering patterns within the dataset for both negative and positive ionization modes. Following metabolic pathway analysis based on the differential metabolites using the KEGG database (Fig. 6D), the top five metabolic pathways identified in positive ion mode were retrograde endocannabinoid signaling, biosynthesis of cofactors, necroptosis, beta-alanine metabolism, and pantothenate and coa biosynthesis. In negative ion mode, the key metabolic pathways included HIF-1 signaling pathway, Pentose phosphate pathway, pyruvate metabolism, pantothenate and coa biosynthesis and pyrimidine metabolism. The differential metabolites analyzed through LC–MS/MS processing showed statistical differences among the groups. Heatmap analysis identified 111 and 48 differential metabolites in positive and negative ion modes, respectively (Fig. 6E).

### Integrated analysis of proteomics and metabolomics in HF rats

**Fig. 7:**
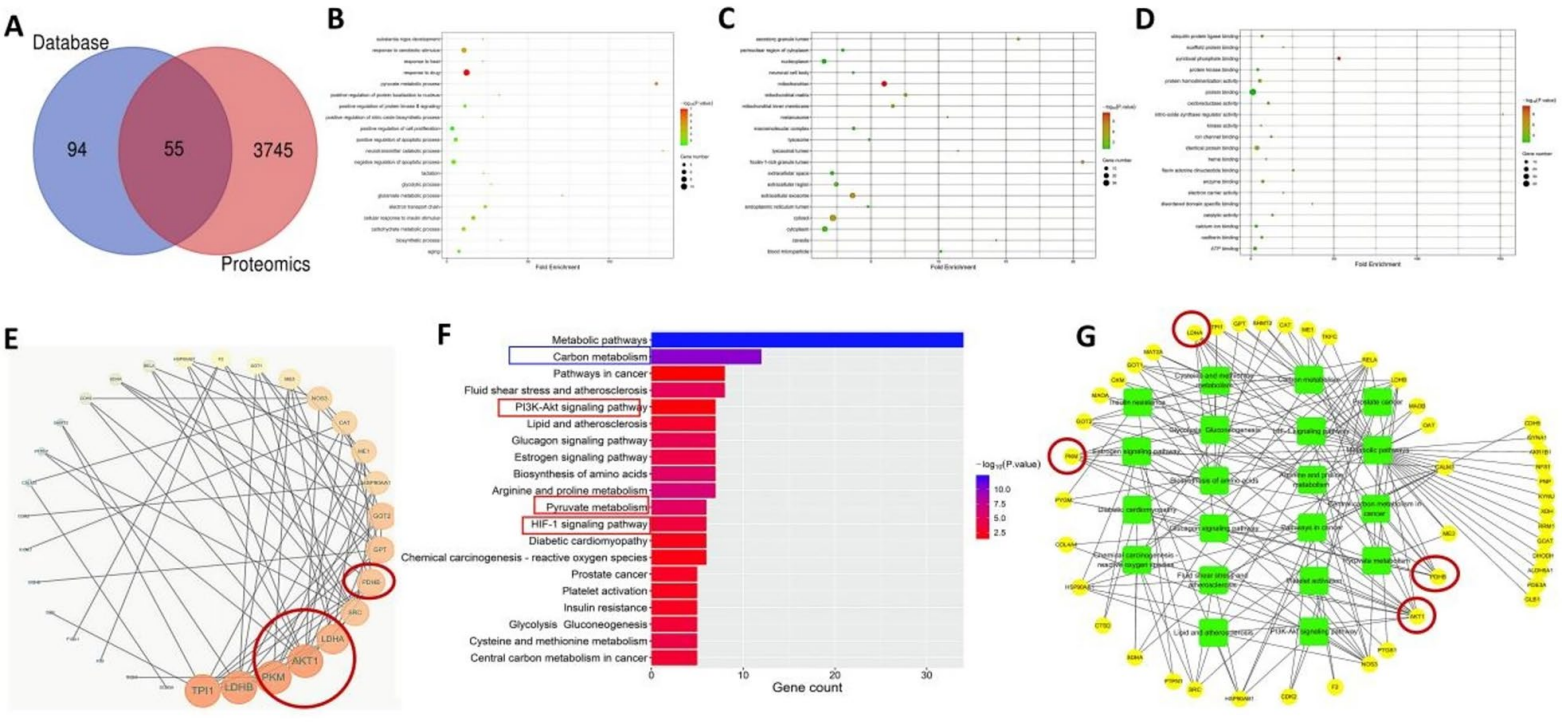
Effects of JX granules on hub genes and critical regulatory pathway by integrated analysis of proteomics and metabolomics. **A** PPI network of HF targets. The colour circles represent the key genes with the different degree values. **B** Top 20 significantly enriched pathways enrichment by KEGG analysis. **C** PPI network of main genes with pathways. The green rectangle nodes represent the pathways and the yellow circle present the relative genes associated with JX granules pharmacologic action. **A** The Venn analysis showed the crosstalk of DEGs in two omics. The GO enrichment analysis, BP (**B**), MF (**C**), CC (**D**). **E** PPI network of HF targets, The colour circles represent the key genes with the different degree values. **F** Top 20 significantly enriched pathways enrichment by KEGG analysis. **G** PPI network of main genes with pathways, The green rectangle nodes represent the pathways and the yellow circle present the relative genes associated with JX granules pharmacological action

To elucidate the relationships between the significantly different proteins and metabolites, an integrative analysis was performed. These key differential genes are prominently displayed in Fig. 7A, which including triosephosphate isomerase 1 (TPI1), lactate dehydrogenase B (LDHB), pyruvate kinase M (PKM), Akt, lactate dehydrogenase A (LDHA), steroid receptor coactivator (SRC), Pyruvate Dehydrogenase Beta (PDHB), etc.

KEGG pathway suggested (Fig. 7B) that proteins of the co-regulated features were mainly involved in the metabolic pathway, carbon metabolism, fluid shear stress and atherosclerosis, PI3K-Akt signaling pathway, glucagon signaling pathway, pyruvate metabolism, HIF-1α signal pathway, etc. To further elucidate the interplay between pathways and hub genes, a pathway network analysis was performed (Fig. 7C). Green squares represented the pathways, while yellow circles denoted the target genes participating in the network. This analysis indicated the critical relationships that underlie the effects of JX granules on HF.

### JX granules improve pyruvate metabolism and the pi3k and HIF-1α signaling pathways

**Fig. 8:**
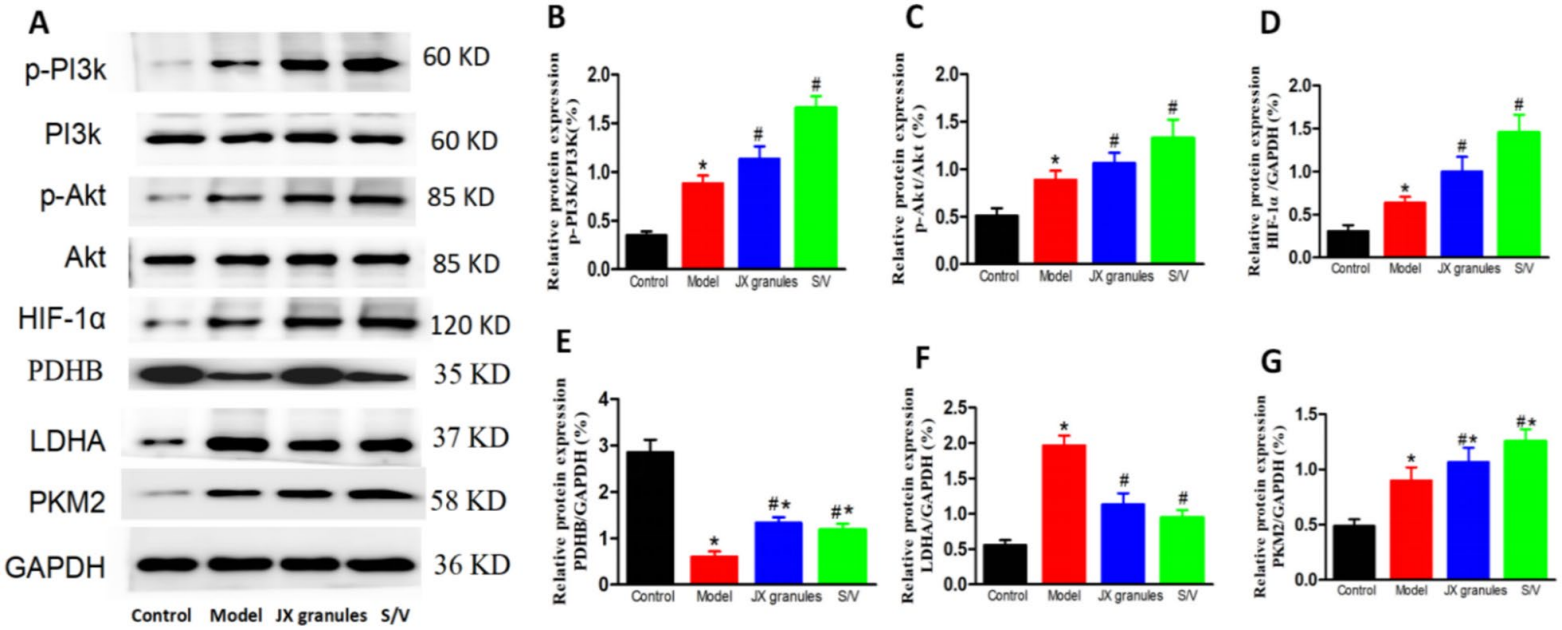
Effects of JX granules on the protein expression of p-PI3K/PI3K, p-Akt/Akt, HIF-1α, PDHB, LDHA, and PKM2. (A-G) The protein expressions of p-PI3K, p-Akt, HIF-1α, PDHB,LDHA and PKM2 were analyzed by western blotting. The protein densitometry was normalized with PI3K, Akt, or GAPDH. And they were quantified of three experiments. ^*^p < 0.05 vs the control group, ^#^ p < 0.05 vs the model group

According to the above results, the pivotal targets influenced by JX granules in HF rats include PDHB, PKM, Akt, LDHA, and HIF-1α, as determined through KEGG pathway analysis. This suggests that JX granules potentially modulate pyruvate metabolism pathway, alongside the PI3K/Akt and HIF-1α signaling pathways. Whether JX granules activate these pathways is verified by western blot analysis (Fig. 8).

In comparison to the control group, p-PI3K/PI3K, p-Akt/Akt, HIF-1α, LDHA, and PKM2 expressions were up-regulated, while PDHB down-regulated in model rats. Treatment with JX granules or S/V further reversed the changes of their expressions. As compared to the model group, p-PI3K/PI3K, p-Akt/Akt, HIF-1α, PDHB, and PKM2 increased by 0.28,0.20, 0.57, 1.21, 0.19-fold, respectively in JX granules, and by 0.87, 0.49, 1.28, 0.96, 0.39-fold in S/V group. LDHA was decreased by 42% in JX granules and 53% in S/V group.

### JX granules increased the production of ATP via enhancing akt activity and reducing apoptosis in h9c2 cells

**Fig. 9:**
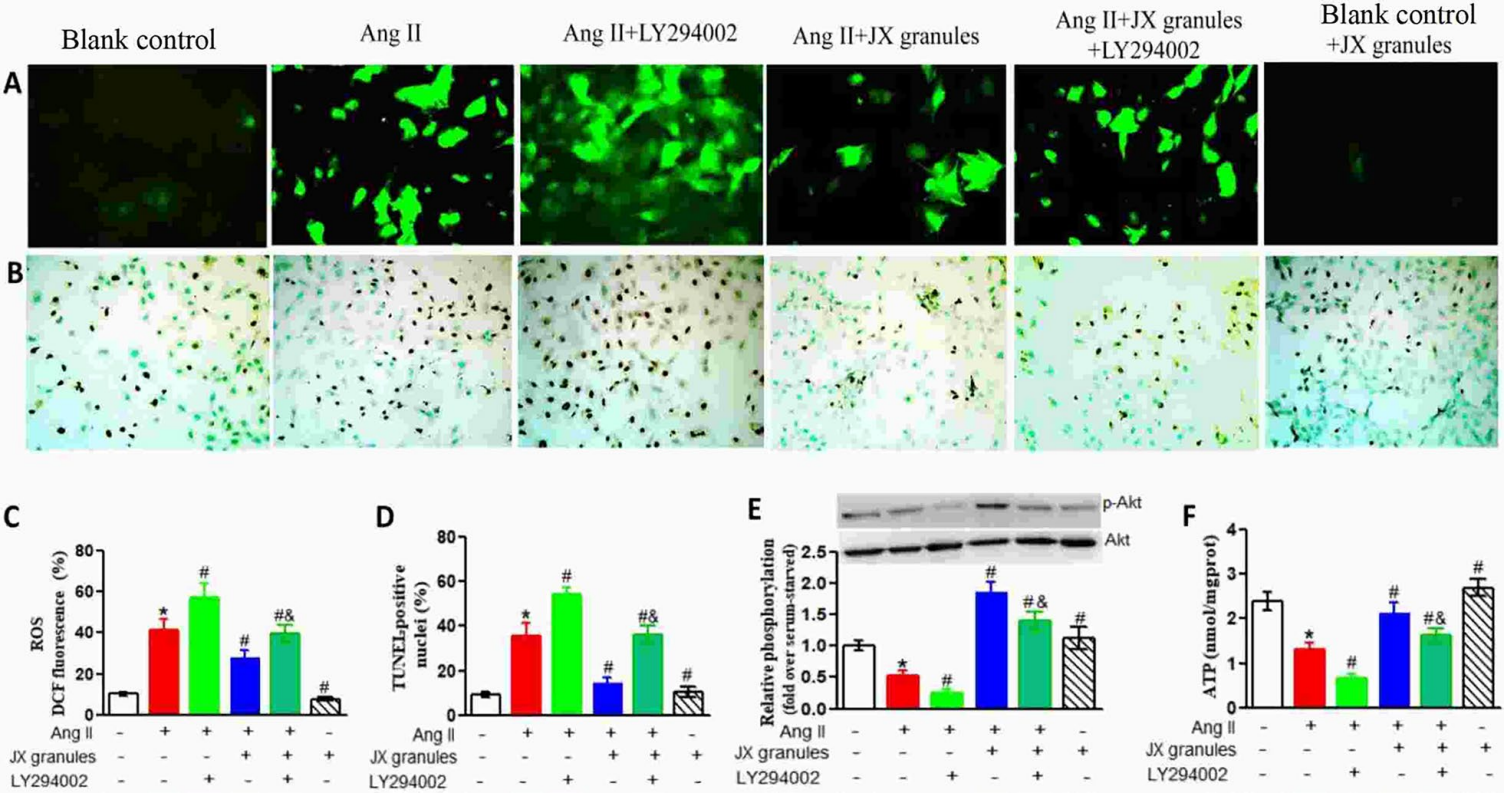
Effects of Jianxin granules on ROS, apoptosis, Akt activity and ATP in response to Ang II incubation. **A** Fluorescence microscopic images of intracellular ROS showed by 2,7′,7-dichlorofluorescein diacetate (DCFH-DA) staining (green, original magnification, × 200). **B** Representative images of TUNEL assay for apoptosis. Brown nuclei indicated cell apoptosis (original magnification, × 200). **C** Corresponding DCF fluorescence value of ROS. **D** TUNEL-positive nuclei were counted in 500 nuclei from random fields per slide and expressed as the percentage of apoptotic cells (apoptotic nuclei/total nuclei × 100%). **E** The expression of Akt and p-Akt were analyzed by Western blotting. Quantification of the ratios of band intensity of p-Akt relative to Akt. **F** The levels of ATP in H9c2 cells. Data are expressed as mean ± SD obtained from three independent experiments. *p < 0.05 compared with blank control. ^#^*p* < 0.05 compared with Ang II challenge cells. ^&^*p* < 0.05 compared with Ang II + LY294002 treated cells

To further confirm that JX granules could inhibit oxidative stress and apoptosis via PI3K-Akt pathways, the specific PI3K inhibitor (LY294002) was used to block this pathway. The H9c2 cells were incubated with Ang II. Then the levels of ROS, the apoptosis index, p-Akt, and ATP were determined (Fig. 9). Ang II stimulation markedly increased ROS fluorescence tensity and apoptosis index in H9c2 cells, which was significantly attenuated by JX granules. Further LY294002 treatment partly prevents the decrease of ROS and apoptosis. Western blot analysis indicated that the activity of Akt markedly decreased in Ang II-incubated cells by 47% when compared with blank control. JX granules treatment markedly increased its activity by 2.5 fold, which can be partially reversed by LY294002 co-treatment. However, JX had no effect on p-Akt activity in the absence of Ang II. The levels of ATP in H9c2 cells incubated with Ang II group were lower than that in the blank control cells. After treatment with JX granules, the levels of ATP increased significantly. Compared with the Ang II + JX granules group, further LY294002 treatment significantly attenuated the production of ATP. JX granules slightly increased ATP in cells without Ang II incubation.

## Discussion

HF is a major clinical syndrome with high prevalence and mortality rates. In this study, proteomics and metabolomics, alongside in vivo and in vitro experiments are employed to explore the effects of JX granules on HF and elucidate their underlying mechanisms. We focus on oxidative stress, proteins related to pyruvate metabolism, and PI3K/Akt-mediated apoptosis. The findings indicate that JX granules enhance LV function and reduce apoptosis in HF rats, which is similar to S/V. JX granules may confer these benefits by augmenting ATP production through the modulation of oxidative stress, mitochondrial function, and protein expression of pyruvate metabolism, and by inhibiting apoptosis via the activation of the PI3K/Akt pathway. This study adds to the limited knowledge regarding the therapeutic effects and underlying mechanisms of JX granules in the treatment of HF. Our findings contribute to the understanding of the potential mechanisms underlying the beneficial effects of JX granules in HF treatment.

### JX granules improved LV function and inhibited cardiac fibrosis

According to the results of echocardiography, HE staining, and Masson staining, it was discovered that JX granules effectively enhanced cardiac function and attenuate cardiac fibrosis in HF, which was similar to S/V. For nearly three decades, JX granules, patented for HF treatment, have displayed remarkable therapeutic efficacy, which was further confirmed by the present study. This formulation encompasses eight herbal constituents: Astragalus Membranaceus, Red Ginseng, Pollen Typhae, Salvia Miltiorrhiza, Polyporus umbellatus, Atractylodes Macrocephala, Cassia Twig, and Semen Lepidii. Astragaloside IV, a crucial compound from Astragalus, is distinguished for its cardioprotective properties, which include mitigating myocardial ischemia, modulating sarcoplasmic reticulum Ca^2+^ transport, promoting angiogenesis, and augmenting energy metabolism [18]. Similarly, ginsenosides from Red Ginseng exhibit antioxidative and anti-apoptotic properties that are beneficial in HF contexts [19]. Pollen Typhae contributes to coronary heart disease management by reducing apoptosis-related pathways, amplifying antioxidant enzymes, and adjusting lipid metabolism [20]. Salvia Miltiorrhiza is recognized for alleviating HF-related inflammation in rodent models [21]. The diuretic efficacy of Polyporus is well-established [22], while Atractylodes Macrocephala shows significant anti-inflammatory, antioxidative, anti-hypertrophic, and anti-fibrotic effects, suggesting their therapeutic potential in cardiovascular disorders [23]. Additionally, Cassia Twig influences ROS production and mitochondrial function [24], and Semen Lepidii aids in ameliorating myocardial ischemia–reperfusion injuries [25]. Therefore, JX granules may exert its anti-heart failure effects through the herbal constituents.

### JX granules prevented mitochondrial injury, attenuated oxidative stress and inhibited apoptosis

Mitochondria are pivotal in various physiological pathways, notably in energy production and the generation of reactive oxygen species. The observation of mitochondrial morphology, depicted by electron microscopy revealed considerable mitochondrial damage within cardiac myocytes of HF rats. Notably, subsequent treatment with JX granules exhibited a restoration of mitochondrial integrity.

Oxidative stress arises due to the excessive accumulation of free radicals and their oxidant metabolites, leading to detrimental effects on subcellular components, such as mitochondrial dysfunction, lipid peroxidation, endoplasmic reticulum stress (ERS), and DNA fragmentation. ROS refers to highly reactive, oxygen-containing molecules or atoms produced by oxidative reactions, such as superoxide anion (${\text{O}}_{2}^{ \cdot - }$), hydroxyl radicals (·OH), and hydrogen peroxide (H_2_O_2_). It is produced during various cellular processes, particularly mitochondrial respiration. Excessive ROS production in myocardium results in oxidative stress, causing damage to cellular structures. MDA is a byproduct of lipid peroxidation and serves as a marker for oxidative stress and lipid damage. Its content reflects oxidative damage to cell membranes. Increased MDA levels signify lipid peroxidation, contributing to membrane instability and cellular dysfunction. T-AOC reflects the overall antioxidant defense capacity, representing the ability to neutralize ROS. Diminished T-AOC levels may indicate a lack of sufficient antioxidant protection. Our findings demonstrate that JX granules effectively alleviate oxidative stress in HF, as evidenced by the reduction in levels of reactive oxygen species (ROS) and malondialdehyde (MDA), along with an increase in total antioxidant capacity (T-AOC). The decrease in ROS levels indicates a reduced burden of oxidative damage in cardiac tissues. Likewise, the decline in MDA levels, a marker of lipid peroxidation, indicates a decrease in oxidative injury to lipids. Moreover, the increase in T-AOC reflects an enhancement in the overall antioxidant defense system. Overall, JX granules promote a more favorable redox balance within the cardiac tissues of HF.

ATP is crucial for cellular energy production and vital for maintaining cardiac function. Damage to the mitochondria results in diminished ATP generation, which is linked to impaired cardiac contractility. Our results indicate that JX granules treatment increases the ATP content in the myocardial tissue of HF. The mechanism could be the active ingredients in JX granules. Several components, such as ginsenoside Rb1 [26] or Astragaloside [27], have been reported to ameliorate energy metabolism in heart failure model.

Apoptosis of myocardial cells plays a pivotal role in HF progression. Caspases, particularly Caspase-9 and Caspase-3, are crucial executioners of apoptosis. And cleaved forms of these caspases indicate their activation. Bax regulates the permeability of the mitochondrial outer membrane, leading to the release of substances from the mitochondria, and ultimately triggering cellular apoptosis. In addition, Bcl-2 exerts an anti-apoptotic effect by regulating the activation of Bax, inhibiting caspase activity, and maintaining calcium homeostasis, thereby safeguarding cells from apoptotic damage. Our results suggested that pro-apoptotic signals in HF rats can be partially attenuated by JX granules treatment.

### JX granules improved pyruvate metabolism

Pyruvate is a key intermediate metabolite derived from glucose metabolism. Under normal conditions, pyruvate is further metabolized in the mitochondria via pyruvate dehydrogenase (PDH) complex to generate acetyl-CoA, which enters the tricarboxylic acid cycle (TCA) for energy production. In HF, however, there is a shift in pyruvate metabolism towards increased lactate production through the upregulation of lactate dehydrogenase (LDH) and impaired PDH activity, favoring glycolytic flux. This reliance on anaerobic metabolism leads to reduced energy production and impaired cardiac function. LDHA, plays a critical role in converting pyruvate to lactate, a process that is enhanced in heart failure. PKM2 is an isoform of pyruvate kinase that promotes the pyruvate conversion to acetyl-CoA, facilitating its entry into the citric acid cycle for energy production. PDHB, on the other hand, is a subunit of pyruvate dehydrogenase complex, which is responsible for the conversion of pyruvate to acetyl-CoA.

The proteomic and metabolomics analyses identified several key differential genes, including TPI1, LDHB, PKM, Akt, LDHA, PDHB, HIF-1, etc. (Fig. 7E). Subsequent western blot analysis confirmed that the administration of JX granules resulted in a downregulation of LDHA expression, indicating a potential suppression of lactate production within cardiac tissues. Additionally, the upregulation of both PKM2 and PDHB in rats treated with JX granules suggests an enhancement in pyruvate utilization and oxidative phosphorylation pathways. Our discovery of JX granules improving pyruvate metabolism in heart failure provides a novel perspective on their potential therapeutic mechanisms.

### JX granules activated pi3k/akt/HIF-1α signal pathway

The PI3K/Akt/HIF-1αpathway stands as a pivotal regulator governing cell survival, metabolism, and angiogenesis. Previous studies have indicated that its dysregulation is associated with impaired cardiac function, increased apoptosis, and altered energy metabolism [28]. While its activation is generally advantageous, as it promotes cell survival, enhances myocardial contractility, and facilitates responses to hypoxia. But excessive activation can result in pathological hypertrophy or arrhythmias [29]. This highlights the importance of maintaining a delicate balance in cardiac pathophysiology to prevent adverse outcomes.

The present proteomics and metabolomics data suggest that Akt and HIF-1 play crucial roles as differential genes (Fig. 7E), and PI3K-Akt and HIF-1α signaling pathways are key regulatory pathways involved in this process (Fig. 7F). Additionally, our results demonstrate that JX granules treatment enhance PI3K and Akt activity in HF rats. And these findings were further supported by cell culture studies showing that JX granules counteracted oxidative stress and apoptosis in H9c2 cells exposed to angiotensin II, suggesting that JX granules activate PI3K/Akt pathway. It was previously found that ginsenoside Rb1 has been shown to improve HF through activation of PI3K/Akt/mTOR pathway [30, 31]. Furthermore, salvianolic acid B and tanshinone IIA in Salvia miltiorrhiza can protect myocardium from ischemia by activating PI3K/Akt dependent pathway [32]. Our study verified that JX granules activated the PI3K pathway, which may be related to the components of astragalus or red ginseng. For the first time, we have employed a multidimensional approach combining omics methodologies, in vivo experiments, and in vitro studies to investigate the potential mechanism of JX granules.

**Fig. 10:**
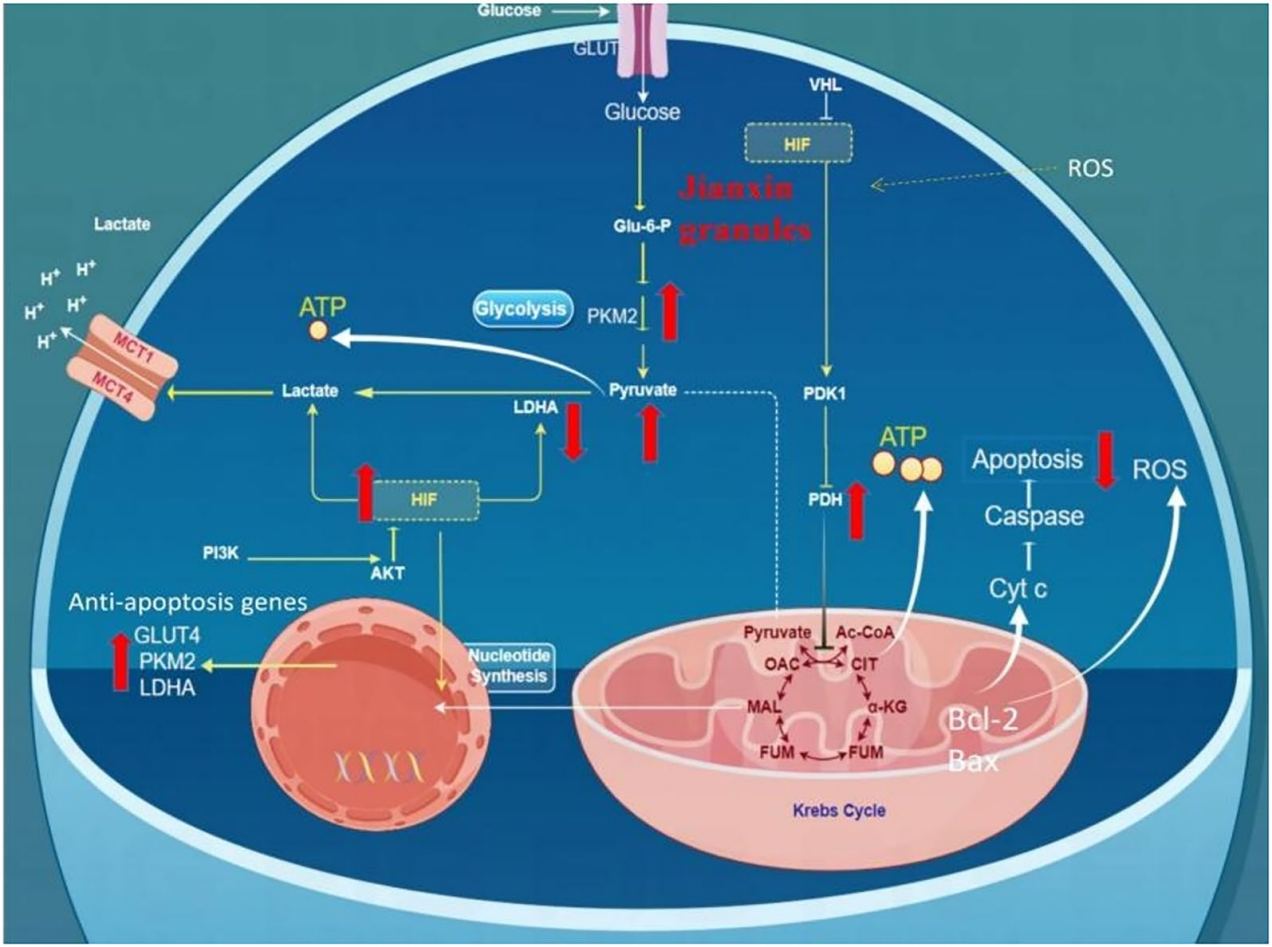
Schematic overview of the mechanism of JX granules in managing heart failure

The downstream molecule HIF-1αplays a significant role in cellular adaptation to hypoxia by promoting the oxygen delivery, angiogenesis, and modulating energy metabolism. In normoxic conditions, HIF-1αprotein undergoes degradation following the hydroxylation of two proline residues. However, under hypoxic conditions, HIF-1αundergoes stabilization, nuclear translocation and dimerization with the β-subunit. This HIF-1α/β complex then regulates the expression of various target genes, including those encoding glycolytic enzymes to facilitate cellular adaptation to hypoxic-ischemic stress [33]. Additionally, HIF-1α activates pyruvate dehydrogenase kinase 1 (PDK1), inactivating PDH and shifting cellular metabolism from aerobic to anaerobic glycolysis. Nevertheless, our results have revealed that JX granules enhance aerobic glucose metabolism by activating HIF-1α. This seems contradictory. One possible explanation is that increased expression of HIF-1α during myocardial ischemia serves as an adaptive response to promote angiogenesis, and then favor mitochondrial respiration and facilitate aerobic glucose metabolism. Besides, it is plausible that JX granules activate PDHB expression and enhance aerobic metabolism through HIF-1α-independent pathways, such as the PPARα/PGC-1α pathway (Fig. 10).

In our study, pyruvate metabolism and the PI3K/AKT signaling pathway were chosen for detailed analysis due to their critical roles in energy metabolism and cellular survival mechanisms, respectively, in the context of heart failure [34, 35]. However, we acknowledge that other pathways, such as galactose metabolism, amino sugar and nucleotide sugar metabolism, and starch and sucrose metabolism, also exhibited significance in our KEGG analysis. These pathways should not be overlooked, and future studies will aim to investigate their roles more deeply, as they may reveal additional insights into metabolic dysregulation in heart failure. We recognize this as a limitation of the present study and aim to address these pathways in subsequent research efforts. Additionally, further research on the rate-limiting enzyme activity of pyruvate metabolism and tricarboxylic acid cycle is necessary to validate these findings. Finally, this study primarily focuses on validating key molecules from the pathways identified through proteomics and metabolomics analysis using partial in vitro experiments. A more detailed exploration of the upstream and downstream pathways will be the focus of our future research.

In summary, our findings indicate that JX granules effectively enhance cardiac function and attenuate apoptosis. Presumably, JX granules mitigate oxidative stress, safeguard mitochondrial integrity, and improve pyruvate metabolism, culminating in an increase in ATP production. Additionally, JX granules exert anti-apoptotic effects by modulating caspases and Bcl-2 family protein expression through the PI3K/Akt signaling pathway. By employing multiple research techniques, the study provides comprehensive molecular insights into HF.

## Conclusion

Our findings indicate that JX granules can substantially enhance LV function and inhibit apoptosis, paralleling the efficacy of S/V. The underlying mechanisms may include the inhibition of oxidative stress and the activation of PI3K/Akt signaling pathway. This research offers valuable insights into the mechanisms that underlie the beneficial effects of JX granules in managing heart failure.

## Supplementary information

Supplementary Material 1

## Data Availability

The mass spectrometry metabolomics data have been deposited to the ProteomeXchange Consortium via the PRIDE partner repository with the dataset identifier PXD048915 and proteomics data from http://www.ebi.ac.uk/pride/archive/projects/PXD048915. It is also available from the corresponding author.

## Abbreviations

ATP: Adenosine triphosphate
Akt: Protein kinase B
BP: Biological process
CC: Cellular component
DEPs: Differentially expressed proteins
HF: Heart failure
JX granules: Jianxin granules
KEGG: Kyoto Encyclopedia of Genes and Genomes
LV: Left ventricular
MDA: Malondialdehyde
MF: Molecular function
PI3K: Phosphatidylinositide 3 kinase
ROS: Reactive oxygen species
S/V: Sacubitril/Valsartan group
T-AOC: Total antioxidant capacity
