# Supplementary material for: Mechanism of jianxin granules in the treatment of heart failure based on proteomics and metabolomics
Source: Chin Med. 2024 Nov 28;19:165. doi: 10.1186/s13020-024-01009-6 (PMC11604013; doi:10.1186/s13020-024-01009-6)
Supplement: Supplementary file 1 — Supplementary Material 1 [file 13020_2024_1009_MOESM1_ESM.docx]

**Supplementary materials**

(1)Proteomics Analysis:

### ****Sample Preparation****:

### For each sample, **2 μg of total peptides** were separated and analyzed.Protein extraction was performed using a lysis buffer with 4% SDS and 100 mM DTT, followed by sonication.

### ****Instrument****:

Proteomics analysis was conducted using a **nano-UPLC (EASY-nLC1200)** coupled to a **Q Exactive HFX Orbitrap** instrument (Thermo Fisher Scientific) with a nano-electrospray ion source.

### ****LC Parameters****:

Chromatographic separation was performed on a **nano C18 column**, with a gradient elution of **water with 0.1% formic acid (A)** and **acetonitrile with 0.1% formic acid (B)**.

### ****Gradient Program****:

The gradient elution program was as follows:

- **0–5 min**: 5% B
- **5–35 min**: 5–35% B
- **35–40 min**: 35–80% B

### ****Mass Spectrometry Parameters****:

The MS/MS analysis was performed using a **Q Exactive HFX Orbitrap** in data-dependent acquisition (DDA) mode and **positive ionization mode**.

- **Resolution**: 60,000 for MS1 and 15,000 for MS2.
- **Scan range**: m/z 300–2000.
- **Collision energy**: 30 eV for higher-energy collisional dissociation (HCD).
- **Spray voltage**: 3.2 kV for both positive and negative modes.
- **Sheath gas flow rate**: 10–40 arb.
- **Capillary temperature**: 320°C.

### ****Software and Data Processing****:

Raw MS files were processed using **Proteome Discoverer (PD) software (Version 2.4.0.305)** with the built-in **Sequest HT search engine**. The MS spectra were searched against the **UniProt FASTA database for Rattus norvegicus (uniprot-Rattus norvegicus-10116-2021-8.fasta)**.

- **Fixed modifications**: Carbamidomethyl [C], TMT Pro (K), TMT Pro (N-term).
- **Variable modifications**: Oxidation (M), Acetyl (Protein N-term).
- **Peptide identification**: Performed with an initial precursor mass deviation of up to 10 ppm and a fragment mass deviation of 0.02 Da.

### ****Quantification and Data Normalization****:

- **Unique peptides** and **Razor peptides** were used for protein quantification, while the **total peptide amount** was used for normalization.
- A **1.5-fold change** was set as the threshold for significance, determined by analyzing inner-quartile data from control experiments using ln-ln plots. The **Pierson’s correlation coefficient (R)** was 0.98, and **95–99%** of the normalized intensities fell between the set fold changes.
- Both **t-tests** and **fold change thresholds** needed to pass to consider the protein significant.

### ****Multivariate Analyses****:

- **2D Hierarchical Cluster Analysis (HCA) HeatMaps** and **Principal Component Analysis (PCA)** plots were performed using **Qlucore Omics Explorer** (Qlucore, Lund Sweden).
- **Gene ontology assignments** and **pathway analysis** were executed using **MetaCore (GeneGO Inc., St. Joseph, MI, USA)**. Interactions identified in MetaCore were manually validated through full-text articles.

**(2)Metabolomics Detection**

· **Instrument**: Thermo Scientific Vanquish UHPLC-Q Exactive system and Thermo Q Precision mass spectrometer

· **Column**: Hyperil Gold C18 column (2.1 mm × 100 mm, 1.9 μm)

· **Mobile Phases**: Not specified directly, but based on common practices, it is likely to be **water with 0.1% formic acid** (A) and **acetonitrile with 0.1% formic acid** (B)

· **Gradient Elution**: Not mentioned in the text, you may need to retrieve the specific gradient from experimental records (it usually involves increasing the percentage of B over time)

· **Flow Rate**: 0.3 mL/min

· **Injection Volume**: 5 μL

**Ionization Mode**: Positive and negative ionization modes

· **Mass Spectrometry Parameters**:

- **Capillary voltage**: Spray voltage of 3.2 kV for both positive and negative modes
- **Cone voltage**: 30 V
- **Source temperature**: Capillary temperature of 320°C (likely equivalent to source temperature)
- **Mass range**: **m/z 100–1200**
- **Sheath gas flow rate**: Set between 10–40 arb
- **Data Acquisition**: **HCD scan** used for data-dependent acquisition (DDA) MS/MS experiments
- **Dynamic Exclusion**: Applied to remove redundant information in MS/MS spectra
